# Evaluation of the Efficacy and Safety of High Dose Short Duration Enrofloxacin Treatment Regimen for Uncomplicated Urinary Tract Infections in Dogs

**DOI:** 10.1111/j.1939-1676.2012.00914.x

**Published:** 2012-04-04

**Authors:** JL Westropp, JE Sykes, S Irom, JB Daniels, A Smith, D Keil, T Settje, Y Wang, DJ Chew

**Affiliations:** Department of Medicine and Epidemiology, University of CaliforniaDavis, CA; Department of Veterinary Clinical Sciences, The Ohio State University, College of Veterinary MedicineColumbus, OH; Bayer HealthCare Animal HealthShawnee Mission, KS

**Keywords:** Amoxicillin-clavulanic acid, Bladder, Enrofloxacin, Lower urinary tract infection

## Abstract

**Background:**

Uncomplicated urinary tract infections (UTI) in dogs usually are treated with antimicrobial drugs for 10–14 days. Shorter duration antimicrobial regimens have been evaluated in human patients.

**Hypothesis:**

A high dose short duration (HDSD) enrofloxacin protocol administered to dogs with uncomplicated UTI will not be inferior to a 14-day treatment regimen with amoxicillin-clavulanic acid.

**Animals:**

Client-owned adult, otherwise healthy dogs with aerobic bacterial urine culture yielding ≥10^3^ CFU/mL of bacteria after cystocentesis.

**Methods:**

Prospective, multicenter, controlled, randomized blinded clinical trial. Enrolled dogs were randomized to group 1 (enrofloxacin 18–20 mg/kg PO q24h for 3 days) or group 2 (amoxicillin-clavulanic acid 13.75–25 mg/kg PO q12h for 14 days). Urine cultures were obtained at days 0, 10, and 21. Microbiologic and clinical cure rates were evaluated 7 days after antimicrobial treatment was discontinued. Lower urinary tract signs and adverse events also were recorded.

**Results:**

There were 35 dogs in group 1 and 33 in group 2. The microbiologic cure rate was 77.1 and 81.2% for groups 1 and 2, respectively. The clinical cure rate was 88.6 and 87.9% for groups 1 and 2, respectively. Cure rates between groups did not differ according to the selected margin of noninferiority.

**Conclusions and Clinical Importance:**

HDSD enrofloxacin treatment was not inferior to a conventional amoxicillin-clavulanic acid protocol for the treatment of uncomplicated bacterial UTI in dogs. Further research is warranted to determine if this protocol will positively impact owner compliance and decrease the emergence of antimicrobial resistance.

Bacterial urinary tract infections (UTIs) have been estimated to occur in approximately 14% of dogs during their lifetime,^1^ with a variable age of onset. The clinical signs of bacterial cystitis include stranguria, pollakiuria, inappropriate urinations, dysuria, and hematuria. Clinically occult UTIs in dogs and cats also have been reported.^2^,^3^ Although there are no studies in dogs to determine the appropriate duration of treatment for simple, uncomplicated UTIs, they usually are treated for 7–14 days.^4^–^6^ Uncomplicated UTI is defined as a UTI without structural, metabolic, or functional urinary tract abnormalities, as well as the absence of other comorbidities, such as cystic calculi, bladder neoplasia, and systemic disorders, such as uncontrolled diabetes mellitus or hyperadrenocorticism^7^ that could favor a more serious adverse outcome. A longer duration of treatment has been suggested for humans^8^ and dogs^6^ with complicated UTIs.

In human patients, uncomplicated UTIs often are treated for a maximum of 3 days; however, single dose protocols also have been described.^9^,^10^ Various antimicrobial regimens for UTI, including fluoroquinolones, have been evaluated; relapsing infections were reported more often in single dose protocols.^10^–^12^ A relapsing or persistent infection occurs when the initial infection was not resolved despite treatment. In contrast, reinfections also may occur, but imply that a new bacterial species or strain infects the patient after a period of sterility.^13^ Efficacy of short duration treatment regimens has been variable, but some higher dose protocols appear to result in longer short- and long-term cure rates.^14^ Fluoroquinolones are commonly used^15^,^16^ because of their spectrum of activity against uropathogens and the high concentrations that are achieved in urine.^17^,^18^ In humans, short durations of treatment and decreased dosing frequency of antimicrobials have been reported to increase compliance, lower costs, decrease adverse effects, and may be as effective as conventional protocols.^7^,^19^

Fluoroquinolones also have been used to treat UTI in dogs. Enrofloxacin^a^ is a fluoroquinolone antimicrobial drug that was developed specifically for veterinary use, and approved dosages range from 5 to 20 mg/kg PO q24h. The efficacy of a high dose, short duration (HDSD) antimicrobial regimen in naturally occurring UTI in dogs has not been evaluated. Therefore, the purpose of this study was to evaluate the efficacy of a 3-day course of HDSD enrofloxacin compared with a 14-day period of treatment with amoxicillin-clavulanic acid^b^ for uncomplicated UTI in dogs. Amoxicillin-clavulanic acid was chosen because this antibiotic is approved for treatment of UTI in dogs, has a spectrum of activity against common uropathogens, reaches high concentrations in the urine, and is commonly prescribed by primary care veterinarians.^4^,^20^ We hypothesized that in naturally occurring uncomplicated UTI, neither microbiologic cure rate nor clinical resolution rate would be significantly different between the 2 treatment groups.

## Materials and Methods

### Study Design

This was a prospective, multicenter, controlled, randomized, and blinded clinical trial. The trial was conducted in accordance with Guidance for Industry #85, Good Clinical Practice.^c^ The study was performed at 2 university teaching hospitals (William R. Pritchard, Veterinary Medical Teaching Hospital at University of California, Davis, and The Ohio State University Veterinary Medical Center) as well as 3 veterinary private practice sites located in Missouri, Louisiana, and Pennsylvania.

Client-owned, adult dogs that weighed 5–50 kg and that were suspected to have an uncomplicated clinical UTI at each study site were eligible for enrollment. Juvenile dogs were excluded from the study because articular cartilage lesions have been reported with the use of fluoroquinolones in rapidly growing dogs.^21^ Young age was defined as <9 months of age for dogs that weighed 5.0–25.9 kg at maturity, <1 year for dogs that weighed 26–45 kg, and <18 months for dogs that weighed >45 kg. A complete medical history was collected. Dogs were excluded from the study if they had a history of persistent or recurrent UTI (defined as >3 UTIs in 1 year with or without a period of sterility), or uncontrolled systemic comorbid diseases that could predispose the dog to recurrent infections. Dogs with diabetes mellitus, hyperadrenocortocism, and urinary incontinence also were excluded. Antimicrobial drug administration within 7 days or corticosteroid administration within 14 days of initial presentation or other compounds containing metal cations or administration of antacids also were criteria for exclusion, the latter because these cations can bind to enrofloxacin and decrease its absorption.^20^

On day 0, a physical examination, which included bladder palpation, was performed on all dogs by 1 of the study investigators. The presence or absence of lower urinary tract signs (LUTS) was recorded. LUTS were defined as pollakiuria, stranguria, and hematuria. A complete blood count (CBC) and serum biochemical analysis also were performed on day 0. In addition, urine was collected by cystocentesis and subjected to urinalysis and quantitative aerobic bacterial urine culture with susceptibility testing by broth microdilution using minimum inhibitory concentration (MIC) testing.

To be enrolled in the study, dogs had to be deemed otherwise healthy based on the history, physical examination, and absence of abnormalities on the CBC and serum biochemical analysis. Ultrasound examination of the urinary bladder was performed only in dogs seen at the university sites. Only dogs with aerobic bacterial urine cultures that yielded ≥10^3^ CFU/mL of at least 1 of the isolates or a mixture of up to 2 target pathogenic bacteria were definitively eligible for inclusion. Pathogenic bacteria were defined as *Escherichia coli, Proteus* spp.*, Klebsiella* spp*., Pseudomonas aeruginosa, Enterobacter* spp*., Staphylococcus aureus, Staphylococcus intermedius* group (*S. pseudintermedius, S. intermedius*, *S. delphini*), alpha-hemolytic *Streptococcus* spp*.,* and *Enterococcu*s spp. Dogs were included if nontargeted bacteria were isolated in conjunction with the targeted pathogens. Dogs with suspected or confirmed pyelonephritis, prostatitis, urinary tract neoplasia, urinary calculi, uncontrolled systemic disease, spinal cord injuries, and suspected CNS disorders were not eligible for enrollment.

Enrolled dogs then were randomized to group 1 or group 2 by random number generation to ensure an equal number of dogs in each group. Dogs in group 1 received enrofloxacin,^a^ 18–20 mg/kg PO q24h for 3 days. Dogs in group 2 received amoxicillin-clavulanic acid,^b^ 13.75–25 mg/kg PO q12h for 14 days. An unblinded veterinary technician enrolled dogs and dispensed medications for each study site. The time of day the antimicrobial was to be administered was not specified to the client. The technician instructed all pet owners not to discuss the treatment regimen with anyone other than the dispenser, but did not otherwise participate in the clinical observations. All veterinarians who performed clinical observations were blinded to treatment group. During the study period, dog owners were asked to record concomitant medications and adverse events that occurred. They were instructed to return their dog to the study site on day 10 (±2 days) and day 21 (±2 days) and bring the prescribed antimicrobial drug with them. During these visits, a complete history was obtained, pills were counted, and a physical examination, urinalysis, and quantitative aerobic bacterial urine culture and susceptibility testing were performed.

Microbiologic cure and clinical cure were evaluated as study endpoints 7 days after discontinuation of antimicrobial treatment, which corresponded to day 10 for group 1 and day 21 for group 2. Long-term microbiologic and clinical cure rates also were determined for group 1 dogs on day 21, 18 days after completing enrofloxacin treatment. Only dogs that had urine cultures performed at those times points were included in the statistical analysis for microbiologic cure. If an animal was removed because of persistent clinical signs, it was considered a clinical failure. Microbiologic cure was defined as a negative aerobic bacterial urine culture, or a culture that yielded <10^3^ CFU/mL of bacteria. Microbiologic failure was defined as an aerobic bacterial urine culture that yielded >10^3^ CFU/mL of the same pathogen as detected in the pretreatment urine specimen. Clinical cure was defined as a lack of any LUTS.

### Culture and Susceptibility Testing

Urine cultures and antimicrobial susceptibility testing were performed at the hospital microbiology laboratories (university sites) or at an independent laboratory^d^ for the private practice sites. Culture and susceptibility testing procedures were performed in a similar manner at each laboratory. Quantitative urine cultures were performed by streaking 5% defibrinated sheep blood and MacConkey agars^e^ each with 10 μL of urine using a calibrated loop. Plates were incubated at 35°C in 5% CO_2_ or ambient air. Organisms were identified using routine microbiologic biochemical testing, including gram stain, spot testing (indole, oxidase), tubed biochemical media, and bacterial identification kits.^f^^22^ Cultures were considered negative when there was no growth after 3 days. Susceptibility testing was performed by broth microdilution according to Clinical Laboratory Standards Institute (CLSI) methods using a commercially available panel of antimicrobial drugs.^g^^23^ Interpretive breakpoints were based on CLSI standards for small animals when available, otherwise standards for human patient isolates were used.^24^

### Statistical Analysis

Noninferiority tests were performed using the Proc Freq procedure^h^ on the dependent variables (proportion of successes) for microbiologic cure as the primary parameter, and clinical outcome as the secondary parameter. The lower bound 1-sided 95% confidence interval was calculated for the success rate difference between group 1 and group 2, and was compared with −20% margin of difference to determine noninferiority.

Body weight, sex, and the results of urinalysis on day 0 were compared between groups. For continuous data, an analysis of variance method was used that included treatment group, sex, the interaction between treatment group and sex, and, where applicable, a baseline covariate. For categorical data, Fisher's exact test was used.

Statistical analysis was performed using a statistical analysis software package^g^ and an alpha level of 0.05 was used for statistical significance. Data were expressed as means ± standard deviations if normally distributed; otherwise, the median and range were provided.

## Results

### Dogs

A total of 92 dogs were evaluated for entry into the study on day 0 across all 5 study sites. Twenty-four dogs ultimately were excluded. Nine dogs were excluded because growth on urine culture was <10^3^ CFU/mL (n = 7), or growth on urine culture was a nontargeted pathogen (n = 2). An additional 15 dogs were excluded after enrollment because either the organism grown on day 10 or day 21 was not the same organism as day 0 (n = 2), owner noncompliance was identified (n = 5), a concurrent disorder that may have predisposed the dog to a UTI was documented (n = 4), the incorrect dosage of medication was prescribed (n = 2), the dog was removed from trial because of an owner-perceived adverse event (n = 1), or a medication was administered that was not allowed according to the study protocol (n = 1).

Of the 68 remaining dogs included in the statistical analysis, 35 (51.4%) were allocated to group 1 and 33 (48.5%) were allocated to group 2. Group 1 was comprised of 30 females and 5 males and group 2 was comprised of 27 females and 6 males. There was no difference in sex distribution between the 2 groups (*P* = .75). All dogs in both groups were spayed or neutered. Group 1 was comprised of 6 Labrador Retrievers, 3 Boxers, 3 mixed breed dogs, 3 Siberian Huskies, 2 Golden Retrievers, 2 Dachshunds, and 1 each of various other breeds. Group 2 consisted of 6 mixed breed dogs, 5 Labrador Retrievers, 3 Dachshunds, 3 Pugs, 2 Golden Retrievers, and 1 each of various other breeds. The median body weight for group 1 dogs was 25.4 kg (range, 5.0–48.8 kg) and for group 2 dogs was 19.0 kg (range, 5.1–50.0 kg) (*P* = .42). The median age for group 1 and group 2 dogs was 7.4 and 9.4 years, respectively (*P* = .42).

No differences in clinical signs were noted between the groups (Table 1). Ten dogs from group 1 and 8 dogs from group 2 had all 3 LUTS. Bladder pain was identified in 6 dogs from group 1 and 7 dogs from group 2.

**Table 1:** Number and percentage of dogs with lower urinary tract signs as reported by owners on day 0. Group 1 dogs were treated with enrofloxacin for 3 days and group 2 dogs were treated with clavulanic acid-amoxicillin for 14 days

Clinical Sign	Group 1 (n = 35)	Group 2 (n = 33)	*P* Value
Pollakiuria	33 (94.3)	33 (100)	.5
Stranguria	21 (60.0)	19 (57.6)	1.0
Macroscopic hematuria	16 (45.7)	14 (42.4)	.8

The mean urine specific gravity of group 1 and group 2 dogs was 1.028 ± 0.012 (range, 1.005–1.050) and 1.026 ± 0.013 (range, 1.002–1.052), respectively. There was no difference in urine specific gravity between the 2 groups (*P* = .92). The median urine pH of group 1 and group 2 dogs was 7.5 (range, 5.0–9.0) and 7.0 ± 1.1 (range, 5.0–8.5), respectively. There was no difference in urine pH between groups (*P* = .72).

**Fig. 1:**
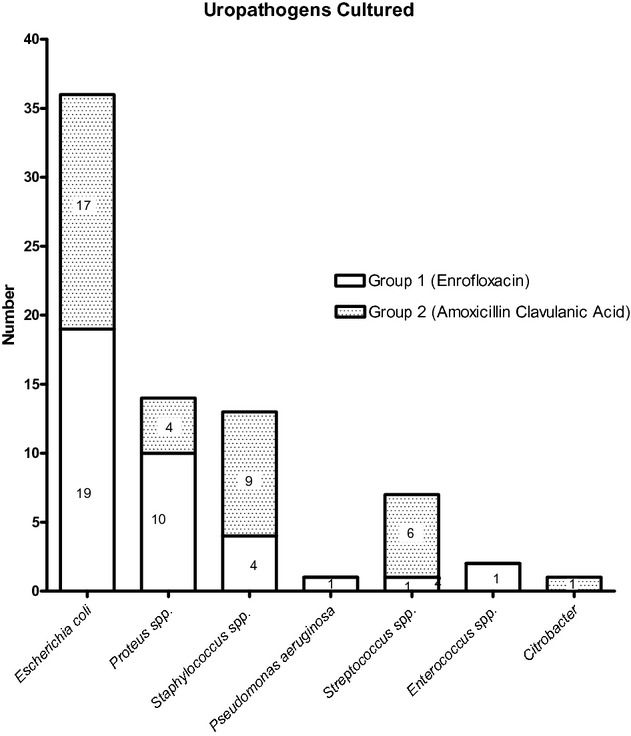
All uropathogens cultured from all dogs at day 0. Group 1 dogs were treated with enrofloxacin for 3 days and group 2 dogs were treated with clavulanic acid-amoxicillin for 14 days.

The most common pathogen isolated from both groups was *E. coli* (Fig 1). Among group 1 dogs, urine culture yielded ≥10^3^ CFU/mL of a single pathogen in 33/35 (94.3%) dogs. In 1 dog, both *E. coli* and a *Staphylococcus* spp. were isolated and in another, both a *Proteus* spp. and *Enterococcus* spp. were isolated. Among group 2 dogs, urine culture yielded ≥10^3^ CFU/mL of a single pathogen in 28/33 (84.8%) dogs. From 5 dogs, significant growth occurred for 2 bacterial species. These were *Staphylococcus* spp*.,* and a *Streptococcus* spp., *E. coli* and a *Proteus* spp., a *Streptococcus* spp. and a *Citrobacter* spp., a *Proteus* spp. and *Staphylococcus* spp., and *S. intermedius* and *Staphylococcus felis*.

### Treatment Efficacy

Only 32 dogs in group 2 were included in the statistical analysis of microbiologic cure because 1 dog was withdrawn by the owner on day 7 because of persistent LUTS, and follow-up urine culture could not be performed. This dog was considered a clinical failure in the analysis of clinical cure (see below). The microbiologic cure rate for dogs in group 1 and group 2 was 27/35 (77.1%) and 26/32 (81.2%), respectively. The lowest end of the 95% confidence interval for this difference was −20.0%, which is equal to the −20% margin of difference and indicated noninferiority of the group 1 treatment compared with that for group 2.

Of the 35 group 1 dogs, 8 (22.9%) had ≥10^3^ CFU/mL bacteria present on urine culture on day 10. Three of these 8 dogs were males. *E. coli* was isolated initially and on day 10 from 5 dogs, and in 3 of those 5 dogs a second organism was present (2 had a *Streptococcus* spp. and 1 an *Enterobacter* spp. infection*)*. One dog each had persistent positive cultures for a *Staphylococcus* spp., *Enterobacteriaceae* spp., and an *Enterococcus* spp. Less than 10^3^ CFU/mL of bacteria were isolated from 2 additional dogs. These dogs were not considered treatment failures according to the study definition. On day 21, 1 of these 2 dogs had a negative culture, and *Enterococcus* spp. was persistently isolated from the urine of the second dog. Two of the dogs that were treatment failures on day 10 (1 dog with an *E. coli* and 1 with a *Enterobacteriaceae* spp.) subsequently were withdrawn from the study and no follow-up cultures were obtained from these dogs on day 21. Only 1 of these 2 dogs had LUTS. Furthermore, an additional dog was withdrawn after day 10 because of persistent LUTS. All of the remaining dogs completed the study. The microbiologic cure rate on day 21 for dogs in group 1 was 22/32 (68.8%).

Of the 32 group 2 dogs that were included in the statistical analysis, 6 (18.8%) had positive urine cultures on day 21. One of these dogs was male. *E. coli* was isolated initially and at day 21 from 4 dogs, and 1 dog each had persistent positive cultures for a *Proteus* spp. and *Staphylococcus* spp. One of the dogs with a positive culture on day 21 had pollakiuria. No other dogs with positive cultures had LUTS.

The clinical cure rate for group 1 and group 2 was 31/35 (88.6%) and 29/33 (87.9%), respectively. The lowest end of the 95% confidence interval for this difference was −12.18%, which was higher than the −20% margin of difference and indicated noninferiority.

Of all the group 1 dogs, 4/35 (11.4%) had LUTS on day 10. All 4 dogs had pollakiuria and 1 of the 4 dogs also had hematuria. Only 1 of the 4 dogs in group 1 with clinical signs on day 10 also had a positive urine culture. The dog with pollakiuria and hematuria had a negative urine culture at day 10, but, as mentioned, was withdrawn from the study and no follow-up culture was obtained on day 21. At day 21, the clinical cure rate for dogs in group 1 was 28/32 (87.5%).

Of all the group 2 dogs, 3/32 (9.4%) had LUTS on day 21. One dog had stranguria, 1 had pollakiuria, and 1 had stranguria, pollakiuria, and pain on bladder palpation. Only the dog with pollakiuria alone had a positive aerobic bacterial urine culture. One additional dog in group 2 also was considered a clinical failure because LUTS recurred and the dog returned for an unscheduled recheck on day 7. Aerobic bacterial urine culture was negative at that time.

### Susceptibility Test Results

Of the group 1 dogs, 3/35 (8.6%) had positive cultures on day 0 for organisms that were resistant to enrofloxacin, and 1 additional dog had a positive culture for an organism with intermediate susceptibility to enrofloxacin. One of these 4 dogs had a positive culture for *E. coli*, and on day 10, this dog had a positive culture for an *E. coli* as well as a *Streptococcus* spp. and was considered a microbiologic failure. On day 21, aerobic bacterial urine culture for this dog was negative despite no additional treatment. The 2nd dog had a positive culture for a *Streptococcus* spp. on day 0, but on day 10, <10^3^ CFU/mL of a *Staphylococcus* spp. were isolated, so this dog was not considered a microbiologic failure. This dog also had a negative bacterial urine culture on day 21. The 3rd dog had a positive culture for *E. coli* on day 0, and on day 10, ≥10^3^ CFU/mL of an *Enterobacteriaceae* were isolated. This was 1 of the 2 dogs withdrawn from the study because of persistent positive culture. The 4th dog had a positive culture for an *Enterococcus* spp. on day 0 with intermediate susceptibility to enrofloxacin. An *Enterococcus* spp. again was isolated (≥10^3^ CFU/mL) on days 10 and 21 with intermediate susceptibility to enrofloxacin. Clinical cures occurred in all 4 of the dogs. All of the other pathogens isolated from dogs in group 1 on day 10 were susceptible to enrofloxacin. When evaluating MIC values for amoxicillin-clavulanic acid for group 1 dogs, only 1 dog had a uropathogen (*Pseudomonas* spp.) that was resistant to amoxicillin-clavulanic acid; this *Pseudomonas* spp. was susceptible to enrofloxacin.

Of the group 2 dogs, 2/33 (6.1%) had positive cultures for organisms on day 0 that were resistant to amoxicillin-clavulanic acid. One of the dogs had a positive culture for both a resistant *E. coli* and a susceptible *Proteus* spp. On day 21, <10^3^ CFU/mL of an unidentified bacteria was isolated from this dog, which was considered a microbiologic cure. Clinical cure also occurred in this dog. The other dog was positive for a *Staphylococcus* spp. on day 0, but had a negative culture on day 10 and day 21 and was free of LUTS. All of the bacteria isolated from dogs on day 21 were susceptible to amoxicillin-clavulanic acid. When evaluating enrofloxacin MIC results for group 2 dogs, 2 dogs had pathogens resistant to enrofloxacin, and 1 had a pathogen with intermediate susceptibility; all 3 isolates were *Streptococcus* spp. In all 3 dogs, the pathogens were susceptible to amoxicillin-clavulanic acid.

### Adverse Events

For group 1 dogs, owners of 15 dogs reported a total of 22 adverse events. In 4 dogs, the owners reported adverse events on multiple days. Based on the history, the attending clinician decided if the adverse event was related to the antimicrobial administration. The assessment was subjective because diagnostics and serum drug concentrations were not obtained for these dogs. The clinicians deemed 9 of the 22 events to be unrelated to administration of the drug and included such clinical signs as lethargy (n = 3), and 1 report each of inappropriate urination, agitation, constipation, diarrhea, abdominal pain, and a small follicular cyst that ruptured on the dog's dorsum. Eleven events were thought to be possibly (n = 8) or probably (n = 3) related to administration of the enrofloxacin and included vomiting (n = 3), lethargy (n = 3), diarrhea (n = 2), anorexia (n = 1), inappetence (n = 1), and hypersalivation after drug administration (n = 1). In 1 dog with lethargy and inappetence, the attending clinician could not determine if the events were related to administration of the antimicrobial because it remained unclear if the dog had these clinical signs before the study. Another dog that was treated with enrofloxacin was removed from the study on day 2 as a consequence of vomiting that occurred 1 and 3 hours after administration of the antimicrobial drug. The dog also was lethargic and inappetent, but responded well to supportive care.

For the group 2 dogs, owners of 14 dogs reported a total of 21 adverse events. In 2 dogs, the owners reported adverse events on multiple days. Ten of the 21 events were thought to be unrelated to administration of the amoxicillin-clavulanic acid and included anxiety (n = 3), polyphagia (n = 2), urinary incontinence (n = 2), inappropriate urination (n = 1), polydipsia (n = 1), and an epidermal inclusion cyst (n = 1). Ten events were thought to be possibly (n = 8) or probably (n = 2) related to the administration of the antimicrobial and included a decreased appetite (n = 5), vomiting (n = 3), diarrhea (n = 1), and constipation (n = 1). In 1 case, the attending clinician could not determine if the event (vomiting) was related to administration of the antimicrobial because the owner reported the clinical sign >5 days after it had occurred.

## Discussion

In this study, we demonstrated that an HDSD enrofloxacin protocol was not inferior to a 14-day antibiotic treatment regimen using amoxicillin-clavulanic acid. The latter is commonly used in clinical practice for treatment of uncomplicated UTI in dogs.^4^ A total of 77.1% of dogs that received HDSD enrofloxacin had negative urine cultures 7 days after completing antimicrobial treatment compared with 81.2% of dogs receiving the amoxicillin-clavulanic acid protocol. The HDSD enrofloxacin protocol also was not inferior to amoxicillin-clavulanic acid with regard to clinical cure rates.

We chose to study enrofloxacin and amoxicillin-clavulanic acid because both drugs are used frequently in clinical practice, are excreted in the urine, and have good activity against many common urinary pathogens, including those isolated in this study.^25^ Trimethoprim-sulfamethoxazole also is recommended for treatment of UTI in dogs,^6^,^26^ and its use is reported frequently in the human literature.^15^ However, trimethoprim-sulfamethoxazole can cause hypersensitivity reactions in dogs, including keratoconjunctivitis sicca.^27^ A number of studies in humans have evaluated the efficacy of fluoroquinolones for treatment of uncomplicated UTI in women.^15^,^16^ We chose to study 1 of the more commonly used fluoroquinolones, enrofloxacin, which is labeled for veterinary use. A higher dose of enrofloxacin was prescribed because HDSD fluoroquinolone protocols in human medicine were reported in some studies to have slightly higher cure rates than low dose protocols.^14^

Our study only examined dogs diagnosed with uncomplicated UTI, but the pathogens isolated were similar to those previously reported for dogs with UTI, some of which were recurrent or relapsing infections.^13^,^28^–^30^ *E. coli*, *Proteus* spp., and *Staphylococcus* spp. accounted for the majority of our isolates. In contrast to other studies, we did not isolate *Klebsiella* spp. from any of the dogs in our study. However, we would expect either drug to have good activity against most *Klebsiella* spp.^25^ As has been reported by others,^31^ only a single bacterial species was isolated from most dogs in our study.

Group 2 dogs had more gram positive uropathogens cultured and no dog in group 2 had a *Pseudomonas* spp. isolated. This study was randomized, and, although a difference in the distribution of gram positive and gram negative pathogens existed between groups (Fig 1), no difference in the prevalence of *E. coli* was found between groups, which is the most common uropathogen reported in dogs. Clinical and microbiologic outcomes for dogs that had growth of only *E. coli* (18 dogs in group 1 and 15 dogs in group 2) were not different, although the limited sample size did not allow us to test for noninferiority (data not shown). We designed our study as a clinical field trial and chose to evaluate data for all pathogens because evidence for differing treatment outcomes for different pathogens is lacking and other variables, such as the possession of virulence factors by different isolates of the same bacterial species also may have the potential to influence outcome.

For those dogs classified as a microbiologic failure, we were unable to definitively identify relapse because molecular investigations of the pathogens using pulse field gel electrophoresis would be needed^32^ and this testing was beyond the scope of this study. Even susceptibility patterns do not reliably distinguish between relapsing or persisting infections and reinfections, because antibiotic resistance patterns do not reflect the genetic composition of the whole chromosome.^33^

Both groups of dogs in this study contained a high percentage of female dogs, which is similar to previous studies of UTI in dogs.^13^,^34^ The microbiologic and clinical cure rate for both groups was similar to short-term cure rates reported for women with uncomplicated UTI when given ciprofloxacin administered as a 250 or 750 mg single dose,^35^ which resulted in microbiologic cure rates of 81.1 and 82.6%, respectively, and clinical cure rates of 89 and 92%, respectively. However, an absence of clinical signs did not imply negative culture results in our study, and likewise, the presence of clinical signs did not predict a positive culture after antimicrobial treatment was instituted. It appears that even in dogs suspected to have uncomplicated UTI, urine culture after antimicrobial treatment may be indicated to be certain that bacteria have been eradicated.

Few studies have evaluated the efficacy of any dosing regimen of antimicrobial treatment for UTI in dogs. No studies of short duration oral antimicrobial protocols for treatment of naturally occurring canine UTI have been published. One-day and 3-day duration treatment protocols were evaluated in an induced *E. coli* canine cystitis model using either injectable amikacin or oral trimethoprim-sulfadiazine.^36^ One-day treatment with either of these drugs was not effective in effecting bacteriological cure after treatment was stopped. The 3-day treatment protocol using trimethoprim-sulfadiazine resulted in bacteriologically sterile urine 14 days after treatment in 4 of 4 female dogs, but in none of 4 male dogs. Cefovecin has been evaluated for the treatment of uncomplicated UTI in dogs when given as a single subcutaneous injection of 8 mg/kg of body weight.^30^ Although this treatment was reported to be effective, it is difficult to compare the bacteriological results in this study to those in our study because different time points and methods of urine collection were used, as well as different definitions for microbiologic or clinical cure. Furthermore, cefovecin is not currently approved for the treatment of UTI in dogs in the United States.

In human medicine, short-duration antimicrobial protocols increase compliance, and result in lower costs of treatment.^19^ In our study, owners were required to keep a log of the drugs administered and compliance was good in both groups we evaluated, although participation in a study may have increased owner compliance. Further studies would be required to determine if owners who use short-duration protocols are more inclined to administer all medications prescribed.

In our study, 91.4% of the isolates from dogs in group 1 were susceptible to enrofloxacin and 93.9% of the isolates from dogs in group 2 were susceptible to amoxicillin-clavulanic acid. The microbiologic and clinical cure rate in our study was lower than that predicted by the organisms' susceptibility for dogs receiving either of the drug regimens used in this study. When evaluating all dogs with persistent positive cultures, none had pathogens resistant to the antimicrobial originally prescribed. The reasons for these differences remain unclear. Although all dogs were screened carefully for underlying diseases that could result in complicated UTI, not all dogs had bladder imaging studies performed and it is possible some dogs with more complicated infections could have been included in the study. Studies that investigate the resistance patterns of urine bacterial isolates from dogs for which the doses of the drugs administered and owner compliance are evaluated also are warranted.

All potential adverse events were reported by owners, but it was up to the attending to clinician to decide if the event was associated with the antimicrobial administered. Adverse events were minimal and only 1 dog that received HDSD enrofloxacin was removed from the study because of gastrointestinal signs. One dog in group 2 had persistent inappetence during treatment, but remained in the study. All other owners felt adverse effects encountered with either antimicrobial protocol were not severe enough to warrant removal from the study.

In summary, an HDSD enrofloxacin protocol was not inferior when compared with a standard antimicrobial regimen using amoxicillin-clavulanic acid for uncomplicated UTI in dogs. The drug regimen was well tolerated by most dogs and can be considered as an alternative for treating uncomplicated UTIs in dogs. Because antimicrobial resistance of canine uropathogens to fluoroquinolones has been reported,^37^,^38^ further research is warranted to determine what impact this protocol will have on the selection for antimicrobial-resistant bacteria. Additional research also should be undertaken to determine if a shortened protocol will positively impact owner compliance. Subsequent studies can help develop guidelines for antimicrobial dosing protocols for uncomplicated UTI.

## Footnotes

a Baytril®, Bayer HealthCare LLC, Animal Health Division, Shawnee Mission, KS

b Clavamox®, Pfizer Animal Health, New York, NY

c VICH GL9, July 1, 2001

d Microbial Research Incorporated, Fort Collins, CO

e Hardy Diagnostics, Santa Maria, CA

f API 20 Strep® and API20E®, bioMérieux, Durham, NC

g ^e^CMV1BURF Plates, Trek Diagnostic Systems, Westlake, OH

h SAS Institute Inc, Cary, NC

## Abbreviations

CFU: colony forming units
CLSI: Clinical Laboratory Standards Institute
CNS: central nervous system
HDSD: high dose short duration
LUTS: lower urinary tract signs
MIC: minimum inhibitory concentration
UTI: urinary tract infection
